# Influential Factors for and Outcomes of Hospitalized Patients with Suicide-Related Behaviors: A National Record Study in Taiwan from 1997–2010

**DOI:** 10.1371/journal.pone.0149559

**Published:** 2016-02-22

**Authors:** Yu-Wen Lin, Hui-Chuan Huang, Mei-Feng Lin, Meei-Ling Shyu, Po-Li Tsai, Hsiu-Ju Chang

**Affiliations:** 1 School of Nursing, College of Nursing, Taipei Medical University, Taipei, Taiwan; 2 Department of Nursing, National Cheng Kung University, Tainan, Taiwan; 3 Division of Colon and Rectal Surgery, Department of Surgery, Mackay Memorial Hospital, Taipei, Taiwan; University of Vienna, School of Psychology, AUSTRIA

## Abstract

**Background:**

Investigating the factors related to suicide is crucial for suicide prevention. Psychiatric disorders, gender, socioeconomic status, and catastrophic illnesses are associated with increased risk of suicide. Most studies have typically focused on the separate influences of physiological or psychological factors on suicide-related behaviors, and have rarely used national data records to examine and compare the effects of major physical illnesses, psychiatric disorders, and socioeconomic status on the risk of suicide-related behaviors.

**Objectives:**

To identify the characteristics of people who exhibited suicide-related behaviors and the multiple factors associated with repeated suicide-related behaviors and deaths by suicide by examining national data records.

**Design:**

This is a cohort study of Taiwan’s national data records of hospitalized patients with suicide-related behaviors from January 1, 1997, to December 31, 2010.

**Participants:**

The study population included all people in Taiwan who were hospitalized with a code indicating suicide or self-inflicted injury (E950–E959) according to the International Classification of Disease, Ninth Revision, Clinical Modification.

**Results:**

Self-poisoning was the most common method of self-inflicted injury among hospitalized patients with suicide-related behaviors who used a single method. Those who were female, had been hospitalized for suicide-related behaviors at a younger age, had a low income, had a psychiatric disorder (i.e., personality disorder, major depressive disorder, bipolar disorder, schizophrenia, alcohol-related disorder, or adjustment disorder), had a catastrophic illness, or had been hospitalized for suicide-related behaviors that involved two methods of self-inflicted injury had a higher risk of hospitalization for repeated suicide-related behaviors. Those who were male, had been hospitalized for suicide-related behaviors at an older age, had low income, had schizophrenia, showed repeated suicide-related behaviors, had a catastrophic illness, or had adopted a single lethal method had an increased risk of death by suicide.

**Conclusions:**

High-risk factors should be considered when devising suicide-prevention strategies.

## Introduction

Suicide is the second major cause of death among adolescents and adults in Taiwan [1] and is a crucial health challenge worthy of thorough investigation. Suicide is a complicated term and there is no single accepted definition. According to Silverman et al., suicide-related behaviors are self-inflicted, potentially injurious acts for which there is evidence (either explicit or implicit) that either (a) an individual wished to use the behaviors of intending to kill himself/herself in order to attain some other end, or (b) an individual intended at some undetermined or known point to kill himself/herself [2]. Suicide-related behaviors can result in no injuries, injuries, or death. Suicide-related behaviors comprise self-harm, self-inflicted unintentional death, undetermined suicide-related behaviors, self-inflicted death with undetermined intent, suicide attempts, and suicide. This study partially adopted the definition from Silverman et al. because it covers more dimensions of suicide and is more suitable for research [2]. However, because the study used hospitalized patients with suicide-related behaviors from national data, subjects without injuries were not included in this study.

According to the World Health Organization (WHO), a suicide attempt is estimated to occur, on average, once every 3 seconds, and one suicide death occurs every minute [3]. Previous studies have shown that 92.3% of suicides attempted were completed during the first or second attempts [4]. The likelihood of death from suicide among those who attempt suicide is relatively high [5]. Suicide attempts, especially repeated suicide attempts, are associated with high risks of death [2, 6]. Therefore, investigating factors related to suicide-related behaviors is crucial for preventing suicides.

Numerous factors contribute to suicide-related behaviors, including gender, socioeconomic characteristics, physical illnesses, and psychiatric disorders. In most countries, suicide rates are higher for men, whereas attempted suicide rates are higher for women [7]. Low income and inactivity are also associated with the overall rates of suicide attempts [8]. A statistical analysis conducted by the Taiwan Suicide Prevention Center showed that disease factors accounted for 8.03% of suicides in 2011, and this proportion increased with age [9]. Previous studies have suggested that certain physical illnesses may increase rates of depression and suicidal ideation [10, 11]. This elevated risk progressively increases depending on the frequency and recentness of hospitalization and is significantly related to all types of diseases [12].

Previous studies have verified that suicide is associated with psychiatric disorders [13], such as schizophrenia, major depressive disorder, and adjustment disorders [14, 15, 16]. In particular, researchers have explored the association between major depressive disorder and suicide [16]. Relevant research has linked suicide attempts to personality disorders [17, 18]. These studies have found that repeated suicidal behaviors are associated with severe personality psychopathological profiles; however, no association was found between the severity of personality disorders and the severity/lethality of suicide attempts among those who attempt suicide [17]. In addition, alcohol-related problems are common among suicide decedents in the absence of psychiatric comorbidities. Individuals with mental health conditions in conjunction with alcohol problems have the highest probability of surviving among those who previously attempted suicide [19, 20]. Psychiatric disorders, gender, socioeconomic status, and physical illnesses are associated with an increased risk of suicide [11, 21]. Most studies have typically focused on the separate influences of physiological or psychological factors on suicide attempts or death by suicide, and have rarely used national data records to explore or compare the effects of major physical illnesses, psychiatric disorders, and socioeconomic status on the risk of repeated suicide attempts and death by suicide.

This study, therefore, used a nationwide database of hospitalized patients with suicide-related behaviors between 1997 and 2010 as an index to concurrently analyze characteristics and methods of patients with suicide-related behaviors and influential factors for repeated suicide behaviors and death by suicide. Specifically, the risk factors for death by suicide and hospitalization for repeated suicide behaviors were examined and compared. The two major research purposes were as follows:

1. What were the characteristics and suicide methods of patients with suicide-related behaviors?
2. What were the influential factors for repeated suicide-related behaviors and death by suicide?

## Methods

### Data source

On March 1, 1995, the National Health Insurance (NHI) was established in Taiwan, covering over 99% of Taiwanese citizens. The NHI in Taiwan is a compulsory health insurance program. Each year, the NHI Administration, Ministry of Health and Welfare, Republic of China (Taiwan) provides data containing encrypted personal identification to the Bureau of NHI to supplement the data in the Taiwan NHI Research Database (NHIRD) for public access. The NHI comprises a wide range of medical services such as inpatient, outpatient, emergency, and traditional Chinese medicine. This study used the “inpatient expenditures according to admissions” data source from the NHIRD [22].

### Inclusion criteria

The study population included all people in Taiwan who were hospitalized from January 1, 1997, to December 31, 2010, with a code of suicide or self-inflicted injury (E950-E959) according to the International Classification of Disease, Ninth Revision, Clinical Modification (ICD-9-CM). The codes are provided below.

- E950 Suicide and self-inflicted poisoning by solid or liquid substances
- E951 Suicide and self-inflicted poisoning by gases in domestic use
- E952 Suicide and self-inflicted poisoning by other gases and vapors
- E953 Suicide and self-inflicted injury by hanging strangulation and suffocation
- E954 Suicide and self-inflicted injury by submersion (drowning)
- E955 Suicide and self-inflicted injury by firearms air guns and explosives
- E956 Suicide and self-inflicted injury by cutting and piercing instrument
- E957 Suicide and self-inflicted injuries by jumping from high place
- E958 Suicide and self-inflicted injury by other and unspecified means
- E959 Late effects of self-inflicted injury

Only hospitalized patients who were at least 12 years old were included when the index hospitalization was begun. Cases in which hospitalizations for suicide-related behaviors exceeded one were considered repeated suicide-related behaviors, and those in which patients with suicide related behaviors died during hospitalization were regarded as death by suicide.

The following information was obtained for each person from the inpatient expenditures according to the admissions category: sociodemographic characteristics (e.g., gender, age during hospitalization for suicide-related behaviors, and economic status) and disease history (e.g., diagnoses of various mental disorders and catastrophic illnesses). According to the National Health Insurance policy in Taiwan, enrollees who qualify as low income earners with monthly, salary-based income ranging from TWD$10,869–14,794 (USD$362–493) are excused from copayments for inpatient or outpatient care. Therefore, patients with certification of waived medical copayment in our study were identified as having low income. The diagnoses of various psychiatric disorders included schizophrenia, bipolar disorder, major depressive disorder, personality disorders, alcohol-related disorders, and adjustment disorders, according to the ICD-9-CM codes that corresponded to the Diagnostic and Statistical Manual of Mental Disorders, Fourth Edition, Text Revision (DSM-IV-TR) [23]. Catastrophic illnesses were defined according to the Ministry of Health and Welfare guidelines and classified into 30 categories such as cancer, chronic mental disorders, and chronic renal failure.

### Data analysis

This study adopted repeated suicide-related behaviors and death by suicide as dependent variables, and sociodemographic variables (i.e., gender, age during hospitalization for suicide attempts, and economic status), catastrophic illnesses, the psychiatric disorders described earlier, and suicide methods (i.e., single or multiple, and main suicide method) as independent variables. First, descriptive statistics were used to determine the characteristics and methods of the patients with suicide-related behaviors. The analyses were performed to explore the following research questions. What was the number of times of hospitalization for people who had suicide-related behaviors in Taiwan during 1997–2010? What were the comprehensive characteristics associated with suicide-related behaviors, including the sociodemographic characteristics of people who exhibited suicide-related behaviors, their disease history, and whether single or multiple methods were used for suicide? What were the main methods used for suicide among hospitalized patients with suicide-related behaviors and death by suicide?

We then used a logistic regression to determine the univariate effects of the sociodemographic characteristics of people who exhibited suicide-related behaviors, their disease history, and the number of methods used for suicide-related behaviors and death by suicide, to ascertain the potential confounding factors for sociodemographic variables.

Second, we modeled the data based on a multivariate logistic model to determine the individual effect of the predictors including catastrophic illness, psychiatric diagnosis, and suicide method on the repeated suicide-related behaviors and deaths by suicide. In addition, the effects of gender, age during hospitalization for suicide-related behaviors, and economic status were also adjusted in the models. Data analyses were conducted using SPSS 19.0 software for Windows (SPSS, Chicago, IL, USA) by using *p* < 0.05 as the statistical significance level.

### Ethical considerations

This study was based in part on data obtained from the NHIRD provided by the Bureau of NHI, Ministry of Health and Welfare and managed by the National Health Research Institutes (NHRI). As required by NHRI regulations, all electronic data were confidential and encrypted for any public requests for research. Although informed consent is unnecessary because of the encrypted data, approval for the study was obtained from Taipei Medical University during May 1, 2012–December 3, 2013.

## Results

### Characteristics of persons who exhibited suicide-related behaviors and suicide methods

Between 1997 and 2010, 57,874 persons were hospitalized for suicide-related behaviors. Prior to December 31, 2010, a total of 55,281 persons who exhibited suicide-related behaviors survived, and 2,593 who had been hospitalized for suicide-related behaviors had died, of whom 2,363 (mostly male) had died in their first suicide attempt. Of the 55,281 suicide survivors, 50,012 persons were hospitalized once for suicide-related behaviors, and 5,269 were hospitalized more than twice because of repeated suicide-related behaviors. The highest number of hospitalizations for a repeated suicide-related behaviors case was 39 (Table 1).

**Table 1:** Number of hospitalizations of people who exhibited suicide-related behaviors in Taiwan, 1997–2010.

No. of times hospitalized	Deaths by suicide (*n* = 2,593)			Still alive on Dec. 31, 2010 (*n* = 55,281)			Total (*n* = 57,874)		
	Male	Female	Total	Male	Female	Total	Male	Female	Total
1	1,474	889	2,363	22,519	27,493	50,012	23,993	28,382	52,375
2	97	80	177	1,687	2,354	4,041	1,784	2,434	4,218
3	13	20	33	289	513	802	302	533	835
4	4	7	11	73	145	218	77	152	229
5–9	4	5	9	65	127	192	69	132	201
10–19	0	0	0	5	8	13	5	8	13
20–29	0	0	0	0	2	2	0	2	2
39 (max.)	0	0	0	0	1	1	0	1	1

Regarding suicide methods (Table 2), most patients used a single method. In particular, self-poisoning was the most commonly used method and was more commonly adopted by women than by men. Hanging was the second most frequently used method. However, cutting, gassing, hanging, jumping, and using firearms were used more frequently by men than by women. According to hospitalization records for patients with suicide related behaviors, 1,450 persons simultaneously used two suicide methods. Most of those who had been hospitalized for suicide-related behaviors with an outcome of death had used a single suicide method. A few people who had died by suicide had used multiple methods; for men, self-poisoning and cutting were the most frequently used methods, and for women, self-poisoning and gassing were the most frequently used methods.

**Table 2:** Methods for suicide-related behaviors and deaths by suicide.

**Methods for suicide-related behaviors (*n* = 57,874)**	**No. of cases (%)**		
	**Male**	**Female**	**Total**
Single method (97.49%)			
Poisoning	15,304 (59.69%)	21,639 (70.29%)	36,943 (65.47%)
Domestic gas	149 (0.58%)	99 (0.32%)	248 (0.44%)
Gassing	2,173 (8.48%)	1,511 (4.91%)	3,684 (6.53%)
Hanging	735 (2.87%)	512 (1.66%)	1,247 (2.21%)
Drowning	112 (0.44%)	242 (0.79%)	354 (0.63%)
Firearms	73 (0.28%)	16 (0.05%)	89 (0.16%)
Cutting	4,577 (17.85%)	5,092 (16.54%)	9,669 (17.14%)
Jumping	563 (2.20%)	617 (2%)	1,180 (2.09%)
Unspecified means	1,682 (6.56%)	696 (2.26%)	2,378 (4.21%)
Later effects	272 (1.06%)	360 (1.17%)	632 (1.12%)
Single method total	25,640	30,784	56,424
Multiple methods (2.51%)			
Poisoning and gassing	232 (39.32%)	310 (36.05%)	542 (37.38%)
Poisoning and cutting	194 (32.88%)	421 (48.95%)	615 (42.41%)
Others	164 (27.80%)	129 (15%)	293 (20.21%)
Multiple methods total	590	860	1,450
**Methods for deaths by suicide (*n* = 2,593)**	**No. of cases (%)**		
	**Male**	**Female**	**Total**
Single method (98.34%)			
Poisoning	936 (59.69%)	624 (62.8%)	1560 (60.6%)
Domestic gas	11 (0.7%)	4 (0.41%)	15 (0.59%)
Gassing	54 (3.44%)	41 (4.18%)	95 (3.73%)
Hanging	209 (13.33%)	138 (14.05%)	347 (13.61%)
Drowning	18 (1.15%)	28 (2.85%)	46 (1.80%)
Firearms	15 (0.96%)	3 (0.31%)	18 (0.71%)
Cutting	111 (7.08%)	26 (2.65%)	137 (5.37%)
Jumping	94 (5.99%)	69 (7.03%)	163 (6.39%)
Unspecified means	97(6.19%)	30 (3.05%)	127 (4.98%)
Later effects	7 (0.45%)	13 (1.32%)	20 (0.78%)
Single method for deaths by suicide total	1,568	982	2,550
Multiple methods (1.66%)			
Poisoning and Gassing	1 (4.17%)	8 (42.11%)	9 (20.93%)
Poisoning and Cutting	9 (37.5%)	3 (15.79%)	12 (27.91%)
Others	14 (58.33%)	8 (42.11%)	22 (51.16%)
Multiple methods for deaths by suicide total	24	19	43

### Influential factors for repeated suicide-related behaviors and death by suicide

There were 5,499 persons hospitalized more than twice for suicide-related behaviors between 1997 and 2010. Women were associated with significantly increased odds of repeated suicide-related behaviors when compared with men, and men had increased odds of death by suicide when compared with women. Older age was significantly associated with decreased odds of repeated suicide-related behaviors, but increased odds of death by suicide. Specifically, people who were older than 70 years when hospitalized for suicide-related behaviors had the highest risk of death by suicide. Individuals who had been diagnosed with a catastrophic illness had increased odds of repeated suicide-related behaviors and death by suicide, as was also seen in the low income group. The presence of any psychiatric diagnosis significantly increased the odds of repeated suicide-related behaviors and decreased the odds of death by suicide. Schizophrenia was the only psychiatric diagnosis not significantly associated with death by suicide (Table 3).

**Table 3:** Characteristics of people who exhibited suicide-related behaviors in Taiwan, 1997–2010.

	Suicide-related behaviors			Deaths by suicide		
				Alive	Dead	
Independent variables	Single behavior (*n* = 52,375), *n* (%)	Repeated behaviors (*n* = 5,499), *n* (%)	Odds ratio (95% CI)^a^^b^	(*n* = 55,281), *n* (%)	(*n* = 2,593), *n* (%)	Odds ratio (95% CI)^a^^c^
**Gender**						
Male	23,993 (91.5)	2,237 (8.5)	1.00	24,638 (93.9)	1,592 (6.1)	1.978(1.824–2.415)***
Female	28,382 (89.7)	3,262 (10.3)	1.233(1.165–1.304)***	30,643 (96.8)	1,001 (3.2)	1.00
**Age at hospitalization for suicide-related behaviors**						
<20	3,210(90.5)	336(9.5)	1.00	3,499(98.7)	47(1.3)	1.00
20–29	12,913(90.0)	1,442(10)	1.067(0.942–1.209)	14,075(98.0)	280(2.0)	1.481(1.085–2.022)*
30–39	11,660(88.7)	1,490(11.3)	1.221(1.078–1.383)**	12,709(96.6)	441(3.4)	2.583(1.908–3.498)***
40–49	9,100(90.3)	983(9.7)	1.032(0.906–1.176)	9,605(95.3)	478(4.7)	3.705(2.739–5.012)***
50–59	5,243(91.6)	483(8.4)	0.880(0.761–1.018)	5,355(93.5)	371(6.5)	5.158(3.796–7.007)***
60–69	4,346(92.4)	359(7.6)	0.789(0.675–0.922)**	4,345(92.3)	360(7.7)	6.168(4.537–8.387)***
≥70	5,903(93.6)	406(6.4)	0.657(0.565–0.764)***	5,693(90.2)	616(9.8)	8.055(5.970–10.869)***
**Income level**						
Regular income	51,382 (90.5)	5,368 (9.5)	1.00	54,227 (95.6)	2,523 (4.4)	1.00
Low income	993 (88.3)	131 (11.7)	1.263(1.050–1.518)*	1,054 (93.8)	70 (6.2)	1.427(1.117–1.824)**
**Catastrophic illness**						
Yes	4,013 (87.1)	594 (12.9)	1.459(1.332–1.599)***	4,213 (91.4)	394 (8.6)	2.172(1.942–2.429)***
No	48,362 (90.8)	4,905 (9.2)	1.00	51,068 (95.9)	2,199 (4.1)	1.00
**Psychiatric diagnosis**						
Schizophrenia	3,895 (82.4)	834 (17.6)	2.225(2.053–2.412)***	4,528 (95.7)	201 (4.3)	0.942(0.813–1.091)
Without schizophrenia	48,480 (91.2)	4,665 (8.8)	1.00	50,753 (95.5)	2,392 (4.5)	1.00
BD	2,286 (75.9)	726 (24.1)	3.333(3.050–3.642)***	2,926 (97.1)	86 (2.9)	0.614(0.494–0.763)***
Without BD	50,089 (91.3)	4,773 (8.7)	1.00	52,355 (95.4)	2,507 (4.6)	1.00
MDD	8,013 (79.4)	2,084 (20.6)	3.378(3.183–3.585)***	9,821 (97.3)	276 (2.7)	0.551(0.486–0.626)***
Without MDD	44,362 (92.9)	3,415(7.1)	1.00	45,460 (95.2)	2,317 (4.8)	1.00
PDs	1,529 (70.6)	638 (29.4)	4.365(3.961–4.809)***	2,139 (98.7)	28 (1.3)	0.271(0.186–0.395)***
Without PDs	50,846 (91.3)	4,861 (8.7)	1.00	53,142 (95.4)	2,565 (4.6)	1.00
ARDs	3,438 (83.7)	671 (16.3)	1.978(1.812–2.160)***	4,004 (97.4)	105 (2.6)	0.540(0.443–0.659)***
Without ARDs	48,937 (91.0)	4,828 (9.0)	1.00	51,277 (95.4)	2,488 (4.6)	1.00
ADs	3,067 (84.3)	572 (15.7)	1.866(1.699–2.050)***	3,621 (99.5)	18 (0.5)	0.100(0.063–0.159)***
Without ADs	49,308 (90.9)	4,927 (9.1)	1.00	51,660 (95.3)	2,575 (4.7)	1.00
**Methods**						
Single method	51,117 (90.6)	5,308 (9.4)	1.00	53,875 (95.5)	2,550 (4.5)	1.00
Multiple methods	1,258 (86.8)	191 (13.2)	1.462(1.252–1.707)***	1,406 (97)	43 (3)	0.924(0.805–1.061)

Abbreviations: CI, confidence interval; BD, bipolar disorder; MDD, major depressive disorder; PDs, personality disorders; ARDs, alcohol-related disorders; ADs, adjustment disorders.

^*a*^ Odds ratio of 1.00 indicates reference group.

^*b*^ The reference category is single behavior.

^*c*^ The reference category is alive.

* *p* < 0.05;

** *p* < 0.01;

*** *p* < 0.001.

After adjustment for the effects of sociodemographic factors (i.e., gender, age during hospitalization for suicide attempts, and economic), patients with psychiatric diagnosis were still significantly associated with elevated odds of repeated suicide-related behaviors and decreased odds of death by suicide, except for patients with schizophrenia (with increased odds of death by suicide). Among all psychiatric disorders, schizophrenia was the only one significantly associated with increased odds of death by suicide, after adjustment for sociodemographic factors. Concerning suicide methods, patients who used multiple suicide methods were most likely to be readmitted to the hospital for repeated suicide-related behaviors. Regarding specific suicide methods, those who attempted suicide by hanging, drowning, using firearms, and jumping had a relatively higher risk of death than that of those who attempted suicide by poisoning, after adjustment for sociodemographic factors. The highest risk of death was for those who attempted suicide by hanging during their first suicide attempt, followed by using firearms, jumping, and drowning (Table 4).

**Table 4:** Adjusted odds ratios for repeated suicide-related behaviors and deaths by suicide according to a logistic model (*n* = 57,874).

	Repeated suicide-related behaviors				Deaths by suicide			
	*β e*stimate	AOR^a^^b^^e^	95% CI	*Nagelkerke R*^*2*^	*β* estimate	AOR^a^^c^^e^	95% CI	*Nagelkerke R*^*2*^
**Catastrophic illness**				.009				.067
Yes	0.436	1.546***	1.410–1.694		0.679	1.972***	1.760–2.209	
No	0	1.00	reference		0	1.00	reference	
**Psychiatric diagnosis**								
Schizophrenia	0.787	2.196***	2.023–2.384	.017	0.158	1.171*	1.007–1.363	.060
BD	1.163	3.200***	2.928–3.499	.026	-0.259	0.772*	0.619–0.962	.060
MDD	1.212	3.361***	3.166–3.568	.058	-0.529	0.589***	0.518–0.669	.064
PDs	1.398	4.045***	3.666–4.464	.030	-0.836	0.433***	0.297–0.632	.061
ARDs	0.745	2.107***	1.925–2.306	.014	-0.637	0.529***	0.433–0.646	.062
ADs	0.586	1.796***	1.634–1.975	.011	-2.117	0.120***	0.076–0.192	.069
**Suicide method**				.007				.061
Single method	0	1.00	reference		0	1.00	reference	
Multiple methods	0.335	1.398***	1.197–1.632		0.054	1.056**	0.918–1.214	
**Main suicide method**^d^								.060
Poisoning					0	1.00	reference	
Domestic gas					0.376	1.456	0.859–2.470	
Gassing					-0.380	0.684***	0.558–0.839	
Hanging					1.993	7.340***	6.401–8.418	
Drowning					1.184	3.268***	2.372–4.504	
Firearms					1.874	6.513***	3.850–11.019	
Cutting					-0.911	0.402***	0.339–0.477	
Jumping					1.585	4.879***	4.089–5.822	
Unspecified means					0.155	1.168	0.970–1.405	

Abbreviations: AOR, adjusted odds ratio; CI, confidence interval; BD, bipolar disorder; MDD, major depressive disorder; PDs, personality disorders; ARDs, alcohol-related disorders; ADs, adjustment disorders.

^*a*^ Odds ratio of 1.00 indicates reference group.

^*b*^ The reference category is single behavior.

^*c*^ The reference category is alive.

^*d*^ If two self-injury methods were used simultaneously, the first E-code was adopted.

^*e*^ Adjusted for gender, age at hospitalization for first-time suicide-related behaviors, and income level.

* p < 0.05;

** *p* < 0.01;

*** *p* < 0.001.

## Discussion

### Characteristics of persons who exhibited suicide-related behaviors and suicide methods

This study referenced national records that detail hospitalized patients with suicide-related behaviors from 1997–2010 to concurrently analyze comprehensive factors for suicide-related behaviors. The records showed that 5,499 persons were hospitalized more than once for repeated suicide-related behaviors, and until December 31, 2010, a total of 2,593 persons who had been hospitalized for suicide-related behaviors had died. Several patients had been hospitalized over 10 times during this 14-year period, possibly because the NHI in Taiwan is easily accessible and inexpensive. In general, we found that male decedents outnumbered female decedents in cases of suicide. Moreover, regardless of the age during hospitalization for suicide-related behaviors or economic status, women had a higher risk of hospitalization for repeated suicide-related behaviors, whereas men had a higher risk of death by suicide. Consistently, suicides resulting in death have higher rates for men, whereas rates of attempted suicide are higher for women [7, 21, 22].

People who had been hospitalized for suicide-related behaviors at a young age were at a greater risk of repeated suicide-related behaviors, and those who exhibited suicide-related behaviors at an older age had an increased risk of death by suicide. Previous studies have shown that older patients who attempted suicide exhibited the lowest risk of repeatedly attempting suicide [8, 15] but had the highest risk of death by suicide [15]. Because of limitations regarding the contents of the database, this study categorized a patient’s economic status into either low family income or regular income, and found that those with low family incomes exhibited a higher risk of hospitalization for repeated suicide-related behaviors and death by suicide. Bolton et al. divided income into four levels and found that people with the lowest income level had the highest risk of suicide attempts [8]. Moreover, most domestic and international suicide research has indicated that men and people with low incomes had a high risk of death by suicide.

Regarding suicide methods, patients may use multiple methods such as self-poisoning followed by gassing. In such cases, although the official reason for death may only be gassing, self-poisoning actually enhanced the possibility of death. Most studies on the factors related to suicide deaths have mainly focused on a single lethal method or the method that contributed most substantially to the death. The NHIRD provides two E-codes that can be used for analyzing the impact of multiple suicide methods on suicide success. In addition, previous studies have consistently found that suicide methods differed between people who were male and female. We found that among patients with suicide-related behaviors, most patients used a single method for their first suicide behavior; in particular, self-poisoning was frequently used by women. This study also identified that 2.51% of patients with suicide behaviors concurrently used multiple suicide methods. Several studies have reported that poisoning was the most common suicide approach [7, 22, 15]. By extensively analyzing the suicide methods, we found that few patients with suicide behaviors used highly lethal methods, such as hanging, drowning, using a firearm, or jumping. These methods were primarily used by male patients and exhibited a high risk of death by suicide. The risk of death by suicide attributable to using gas or cutting was significantly low. This result supported the findings of Runeson et al. who used a Swedish linked registry for the period from 1973–2003 and found that regardless of gender, Swedes frequently used self-poisoning and cutting as suicide methods, which were not significantly associated with the outcome of death by suicide; however, hanging, drowning, using firearms, and jumping were significantly correlated with death by suicide [22]. Another study followed those who attempted suicide for 37 years subsequent to their attempt and found that after they indexed the suicide attempts by self-poisoning, suicide deaths continued to accumulate for the following four decades after the index attempt was completed [24]. A history of suicide attempts by self-poisoning is indicative of a high suicide risk over a lifetime [24]. This study used a follow-up period of 14 years to investigate cases of death by suicide. Differences in follow-up times and file types might have been factors causing variations between our results and the results of others. However, similar to numerous domestic and international studies, we showed that most patients used drugs, and although lethal suicide methods were rarely used, the risk of death by suicide when lethal methods were used was considerably high.

### Influential factors for repeated suicide-related behaviors and death by suicide

Numerous suicide studies have emphasized the importance of a mental illness as an influential factor for suicide-related behaviors. This study analyzed the associations of schizophrenia, bipolar disorder, major depressive disorder, personality disorders, alcohol-related disorders, and adjustment disorders with suicide and found that these diseases increased the odds of hospitalization for repeated suicide-related behaviors. In this study, the odds of people with schizophrenia exhibiting repeated suicide-related behaviors were not the highest; however, after the effects of gender, age during hospitalization for suicide-related behaviors, and economic status were adjusted for, the odds of death by suicide in people with schizophrenia were significantly higher than the odds in those diagnosed with other mental disorders. According to the WHO, suicide is defined as “the act of deliberately killing oneself” [3]. Several studies have indicated that the suicidal behavior of people with schizophrenia was influenced by positive (e.g., hallucinations and delusions) and negative symptoms of schizophrenia [13, 25, 26]. A Finnish study indicated that the number of repeated-suicide hospitalizations by people with schizophrenia was higher than that by people with mood disorders. However, another study found that all causes of death were not statistically significant [15]. A Swedish study showed that the hazard ratio for suicides resulting in death in people with schizophrenia was the highest among all mental disorders [27]. These results suggested that schizophrenia may be an evocative factor for suicide and thus should be extensively investigated.

A study found that patients with affective mood disorders (e.g., bipolar disorder and major depressive disorder) possessed a significantly high risk of hospitalization for repeated suicide-related behaviors, but a low risk of death by suicide. Consistently, another previous study found that schizophrenia, mood disorders, personality disorders, and alcohol-related disorders increased the risk of repeatedly attempting suicide, and alcohol-related disorders were significantly correlated with death by suicide [15]. Moreover, people with DSM-IV axis I psychiatric disorders (e.g., bipolar disorder, depressive disorders, schizophrenia, and adjustment disorders) had a significant hazard ratio for a completed suicide [27]. Nock et al. [20] evaluated the suicide rates in 21 developed countries and found that bipolar disorder yielded the highest risk of suicide attempts. In summary, adjustment disorders increase the risk of suicide-related behaviors, supporting the results obtained in this study. However, the adjustment disorders considered in this study yielded a significantly low risk of death by suicide. This result slightly differed from those presented by the Swedish study, potentially because we considered only patients with suicide behaviors who died during hospitalization and could have underestimated the number of patients who died by suicide. Conversely, the Swedish study [27] used hospitalization data obtained from the Swedish register, which contained data on more than 90% of the deaths occurring in Sweden. Another reason for this finding is that the payment of national health insurance in Taiwan is relatively convenient and cheap. Patients with suicide-related behaviors could hospitalize through outpatient or emergency departments under national health insurance coverage. Hence, patients with suicide-related behaviors were more likely to be rehospitalized. In addition, the definition of suicide in our study included a wide range of suicide and self-injurious behavior, indicating that some hospitalized patients might not have intended to die.

Our results showed that patients with adjustment disorders exhibited a significantly high risk of hospitalization for repeated suicide-related behaviors, but possessed the lowest risk of death by suicide. Consistently, a Swedish study [27] found that affective disorders increased the hazard ratio of death by suicide, whereas adjustment disorders or posttraumatic stress disorder did not exert a significant effect. Gradus et al. found that adjustment disorders were significantly correlated with suicidal ideation and suicide attempts, but not with completed suicides [14]. According to the DSM-IV-TR, an adjustment disorder diagnosis requires exhibition of emotional or behavioral symptoms triggered by one or more identifiable stressors [23]. After the suicide attempt, warnings signs for repeated suicide or counseling provided by mental health personnel may mitigate the stress or pressure that those who attempt suicide experience, thereby reducing their risk of death by suicide.

Substance abuse (especially alcohol) is a focal topic of suicide research. This study found that the odds of hospitalization for repeated suicide-related behaviors of patients with alcohol-related disorders were significantly high, but their odds of death by suicide were low. Numerous studies have investigated the association between alcohol abuse and suicide. For example, Sher [28] reviewed studies on alcohol abuse and suicide in 13 countries and found that the suicide rate was positively correlated with alcohol abuse in 10 countries, but no correlation was identified in three countries. Landberg [29] obtained data from the WHO mortality database, and analyzed the correlation between drinking and suicide in seven Eastern European countries. He found a positive correlation between alcohol consumption and suicide rates in six countries (i.e., Russia, Belarus, Poland, Bulgaria, the Czech Republic, and Hungary), but no correlation in Germany. Haukka et al. [15] found that in Finland, a diagnosis of an alcohol-related disorder yielded a significantly high risk of repeated suicide attempts and death that were attributable to other causes. Other studies have indicated that the primary causes of death in patients with alcohol-related disorders were associated with violence. Particularly, the three leading causes of death among patients with alcohol-related disorders were accidents and violence, alcohol poisoning, and acute ischemic heart disease [30]. Based on the aforementioned studies, the association between alcohol abuse and suicide remains questionable. Because alcohol use might cause impulsive or uncontrollable behaviors, deaths from accidental suicide attempts after alcohol intake should be prevented.

The odds of repeated suicide-related behaviors by patients with personality disorders were the highest, followed by the odds of death by suicide for patients with adjustment disorders. Previous studies have found that 74% of those who attempted suicide were patients with personality disorders, and personality disorders were significantly correlated with repeated suicide attempts [17, 21], but not with death [17]. Therefore, several researchers have excluded axis II disorders from their studies on suicides by patients with major depressive disorder, and have found that axis I disorders did not elevate the risk of multiple suicide attempts [18]. People diagnosed with borderline personality disorders or who possessed a comorbidity with personality disorders had a high correlation with repeated suicide attempts [8, 18], indicating that this diagnosis may result in repeated suicide-related behaviors but not death.

Regarding general healthcare status, this study investigated catastrophic illnesses and found that people with a catastrophic illness possessed high risks of hospitalization for repeated suicide attempts and death by suicide. The NHI in Taiwan is considered a type of social welfare insurance, in which patients with cancer, uremia, stroke, or chronic mental illnesses requiring long-term treatment can apply for a catastrophic illness card to reduce their medical expenses. According to the Taiwan Suicide Prevention Center’s statistics (2013), disease factors accounted for more than 50% of suicide causes, and this percentage increased with age. Research has suggested that medical problems increase the risks of depression and suicidal ideation [11, 19]. Several physical illnesses may raise the rates of depression and suicidal ideation [10, 11, 31], and depression and suicidal ideation are critical risk factors for death by suicide [21]. Patients with catastrophic illnesses, such as cancer, have nearly twice the incidence of suicide compared to the general population [31]. In summary, the results of this study corresponded with those of previous studies, demonstrating that patients diagnosed with catastrophic illnesses are prone to suicide-related behaviors.

### Limitations

Some vital limitations should be considered when interpreting these results. First, the NHIRD is primarily used for collating health insurance costs and therefore does not provide sociodemographic information on potential factors related to suicide, such as marital status and life stressors. Although the limitations of the database hindered this study from obtaining data regarding identifiable stressors for each suicide case, we are certain that identifiable stressors exist in patients with adjustment disorders. Furthermore, whether patients intended to kill themselves could not be determined by the data, and injuries undetermined to be accidental or intentional were not analyzed in this study. Second, we analyzed hospitalization data from 1997–2010, excluding suicide hospitalization records before 1997. Moreover, in contrast to another study [32], which recruited all hospitalized patients who had attempted suicide and divided them into those who attempted suicide for the first-time and those who had previously attempted suicide, we identified cases in which we considered patients who had been hospitalized for suicide-related behaviors more than once to exhibit repeated suicide-related behaviors. These selections may have resulted in an underestimation of the number of patients with repeated suicide-related behaviors. Third, this study defined death by suicide as death during hospitalization for suicide-related behavior, which may have underestimated the number of deaths by suicide. Future studies should integrate and compare the data from other medical sources, such as outpatient and emergency departments, for a more comprehensive understanding of how demographic and disease factors affect repeated suicide attempts and death by suicide. Fourth, we lacked a control group with hospitalization but no suicide-related behaviors. Finally, the NHI in Taiwan covers all necessary medical treatments, and most commercial insurance companies refuse to cover hospitalization charges attributable to suicide. Therefore, we assert that the number of suicide hospitalization cases may have been underestimated.

## Conclusions

Overall, the results of this study indicate that people who were female, had been hospitalized for suicide-related behaviors at a younger age, had low income, had a psychiatric disorder (e.g., personality disorder, major depressive disorder, bipolar disorder, schizophrenia, alcohol-related disorder, or adjustment disorder), had a catastrophic illness, or had been hospitalized for suicide-related behaviors that involved two methods of self-inflicted injury had a higher risk of hospitalization for repeated suicide-related behaviors. Those who were male, had been hospitalized for suicide-related behaviors at an older age, had low income, had schizophrenia, exhibited repeated suicide-related behaviors, had a catastrophic illness, or had adopted a single lethal method had an increased risk of death by suicide. We found that for hospitalized patients with suicide related behaviors, the common influential factors for repeated suicide-related behaviors and death by suicide were schizophrenia, low income, and a catastrophic illness. Therefore, this study recommends that the limited resources allocated to suicide prevention should be focused on by considering these risk factors. The early identification of high-risk groups based on these influential factors is crucial. In addition, multidisciplinary medical services—such as the work of physicians, nurses, social workers, and psychologists—should be integrated to provide comprehensive care that addresses physical, psychological, and social problems, as well as to reduce the possibility of repeated suicide-related behaviors and death by suicide.
